# Cerebral abscess secondary to post-esophageal dilation bacteremia: A case report with a pathophysiological association to consider

**DOI:** 10.3389/fped.2026.1805960

**Published:** 2026-04-27

**Authors:** Ana A. Ortiz-Hernández, Alfonso Marhx Bracho, Eduardo Arias de la Garza, G. H. Cristerna Tarrasa, Victor Manuel Pérez Robles, Beatriz Llamosas-Gallardo

**Affiliations:** 1 División de Desarrollo y Enlace Institucional, Instituto Nacional de Pediatría, Ciudad de México, Mexico; 2 Subdirección de Cirugía, Instituto Nacional de Pediatría, Ciudad de México, Mexico; 3 Subdirección de Consulta externa, Hospital del Niño Morelense, Cuernavaca Morelos, Mexico; 4 Departamento de Infectología, Instituto Nacional de Pediatría, Ciudad de México, Mexico; 5 Departamento de Infectología, Hospital del Niño Morelense, Cuernavaca Morelos, Mexico

**Keywords:** bacteremia, Batson plexus, caustic ingestion, cerebral abscess, esophageal dilation

## Abstract

**Background:**

Accidental caustic ingestion is a pediatric emergency that may result in severe esophageal injury and long-term complications, including strictures requiring repeated dilation. Cerebral abscess is a rare but potentially fatal condition in children and has only sporadically been reported following esophageal dilation.

**Case presentation:**

We report the case of a 3-year-old girl who developed an esophageal stricture following accidental ingestion of sodium hydroxide. After multiple esophageal dilations, the patient presented with electrolyte abnormalities and neurological symptoms. Neuroimaging revealed a large left frontotemporal cerebral abscess with mass effect. Surgical drainage was performed, and *Streptococcus intermedius* was isolated from the abscess cultures. Prolonged targeted antimicrobial therapy led to clinical stabilization, although residual neurological deficits persisted.

**Conclusion:**

This case highlights a rare but severe complication associated with repeated esophageal dilation in pediatric patients. Transient bacteremia caused by mucosal trauma, combined with hematogenous dissemination through the Batson venous plexus, may explain cerebral seeding. Increased clinical vigilance in children with fever and early neuroimaging are crucial if any neurological symptoms develop after esophageal dilation.

## Introduction

Accidental ingestion of caustic substances by pediatric patients is a serious and frequent medical emergency worldwide, particularly in those younger than 5 years old, and can result in life-threatening complications, primarily involving the gastrointestinal tract (1–4). Neurological sequelae, such as cerebral abscesses, are exceedingly rare and typically occur in the context of predisposing conditions. We present the case of a cerebral abscess associated with multiple instrumentations for esophageal stricture due to an esophageal burn.

## Case description

On 19 December 2024, a 3-year-and-6-month-old female patient suffered accidental ingestion of 50% sodium hydroxide, which caused a grade IIIA esophageal burn according to Zargar's classification. A Stamm gastrostomy tube was endoscopically placed for esophageal dilation treatment. Over the subsequent months, seven anterograde dilations were performed for severe stricture management. The last two procedures were performed on 2 and 9 April 2025. Furthermore, 24 hours after the dilation on 2 April, she developed self-limited hemoptysis.

She was admitted on 15 April with a diagnosis of complications associated with the dilation, with a 4-day history of high-grade fever (up to 39.5 °C), postprandial vomiting, abdominal pain, cough, and a severe frontal headache, with empirical home treatment with paracetamol resulting in minimal improvement. An esophagogram ruled out an esophageal perforation. Moreover, 24 h after admission, she developed somnolence, hematemesis with a hemoglobin drop of 2 g/dL, and hyponatremia (123 mEq/L). Conservative management based on proton-pump inhibitors, prokinetics, antiemetics, electrolyte correction, and gastrostomy tube drainage was continued, resulting in clinical improvement.

On 18 April, she developed bradycardia, somnolence, recurrent hyponatremia (123 mEq/L), a urinary sodium level of 128 mmol/L, and an increase in C-reactive protein (CRP) of 5.4 mg/L. Thus, the patient was transferred to the Pediatric Intensive Care Unit for 24 h, and conservative management and electrolyte correction were continued, with gradual stabilization. However, irritability, somnolence, and headache persisted during hospitalization. The patient was discharged 13 days later.

During outpatient follow-up, the neurological symptoms persisted, and the patient developed acute right-sided spasticity, paresthesia, and hemiparesis on 10 May.

**Figure 1:**
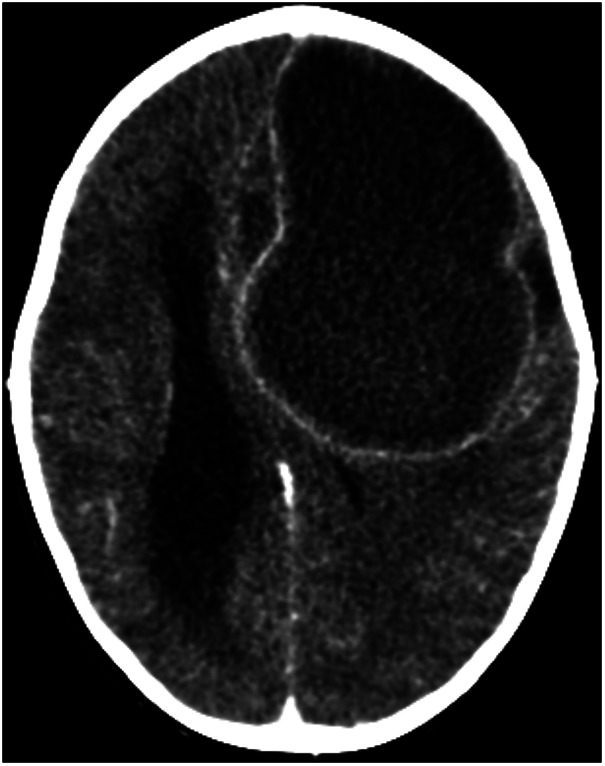
Initial contrast-enhanced cranial computed tomography showing a cerebral abscess. Axial contrast-enhanced cranial computed tomography demonstrates a large left frontotemporal hypodense lesion with peripheral ring enhancement, consistent with a cerebral abscess. Extensive surrounding vasogenic edema is observed, associated with subfalcine herniation and significant midline shift toward the right hemisphere. Compression of the ipsilateral lateral ventricle is also evident.

She was evaluated until 13 May and was then readmitted with somnolence, aphasia, an inability to sit, and a Glasgow Coma Scale (GCS) score of 12 without seizures. Based on the symptoms, a cranial computed tomography (CT) scan was performed, which revealed a left frontotemporal cerebral abscess with perilesional vasogenic edema, subfalcine herniation, and a midline shift (>5 mm left-to-right) (Figure 1). Other causes of the cerebral abscess, such as otitis, sinusitis, dental caries, or cardiac disease, were ruled out. An echocardiogram showed no structural abnormalities or cardiac septic foci.

Empirical antimicrobial therapy with ceftriaxone 100 mg/kg/BID, metronidazole 30 mg/kg/TID, and vancomycin 60 mg/kg/QID was initiated, along with antiepileptic management.

She was transferred to the *Instituto Nacional de Pediatria* for surgical management. Upon admission, she was stable, and her GCS score was 11, with the following laboratory results: Hb, 12.8 g/dL; leucocytes, 16,700 10^3^/µL; CRP 6.53 mg/L, procalcitonin, 1.9 ng/mL; sodium, 128 mEq/L; urinary sodium, 152 mmol/L. A new CT scan confirmed a left frontoparietal abscess, subfalcine herniation, collapse of the left lateral ventricle with contralateral dilation, and diffused cerebral edema with secondary widening of the cranial sutures.

**Figure 2:**
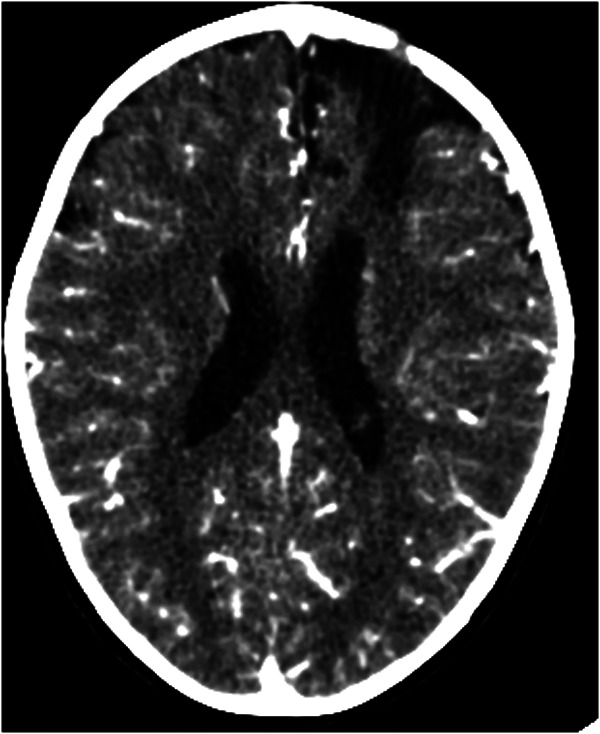
Postoperative contrast-enhanced cranial computed tomography (CT). An axial contrast-enhanced cranial CT scan obtained after surgical drainage of the cerebral abscess shows a reduction of the previously described mass effect, partial resolution of the midline shift, and improved ventricular symmetry. Residual postoperative changes and surrounding edema are observed in the left frontotemporal region.

The patient underwent urgent surgical drainage via a left precoronal burr hole, yielding 1,700 cc of purulent material (Figure 2). Cultures were positive for *Streptococcus intermedius*.

After surgery, targeted antimicrobial therapy was continued for 35 days using the same initial doses, without requiring electrolyte corrections. An immunoglobulin blood test was performed to rule out immunodeficiency, and the following normal values were reported: IgG, 951 mg/dL; IgM, 122 mg/dL; IgA, 94.3 mg/dL; IgE, 20.9 IU/mL. Finally, the patient’s sodium level was 140 mEq/L.

The patient was discharged with a rehabilitation program for her residual right hemibody hemiparesis and continues to require periodic esophageal dilations.

## Discussion

Caustic ingestion remains a relevant clinical and public health issue, even in developed nations. Evidence from Denmark, reported by Christensen et al., documented 102 hospitalizations among children under 16 years of age between 1976 and 1991, corresponding to an annual incidence of 10.8 per 100,000 inhabitants. These findings highlight not only the magnitude of the problem but also the ongoing burden of caustic ingestion in pediatric populations (5).

A systematic review and meta-analysis by Rafeey et al. identified preschool-aged boys as the highest-risk group, with a mean age of 2.78 ± 2.02 years (OR = 0.53, 95% CI, *p* = 0.08), with the most common cause being domestic cleaning products, the most commonly ingested caustic agents, being improperly stored (6).

Injuries associated with caustic ingestion in the upper digestive tract are located at the oral mucosa, pharynx, larynx, and esophagus, while complications such as esophageal stricture occur in 5%–17% of cases, depending on burn severity (1–3). Esophageal dilation, whether via an anterograde or retrograde approach, is the preferred therapeutic procedure to restore esophageal lumen patency and function and requires multiple instrumentations. However, multiple instrumentation is associated with complications such as bleeding, perforation, tracheoesophageal fistula, mediastinitis, and bacteremia (4–10).

Transient bacteremia is an expected consequence of esophageal dilation, resulting from disruption of the mucosal barrier and subsequent translocation of resident oropharyngeal and esophageal microorganisms (11). In the majority of patients, this bacteremia is self-limited and asymptomatic; however, in individuals with predisposing conditions such as structural heart disease, immunosuppression, or prosthetic valves, severe infections—including brain abscesses—may occur. Reported incidence rates of transient bacteremia following dilation range from 22% to 50% and are significantly higher than those associated with other gastrointestinal endoscopic procedures, underscoring the clinical relevance of this complication (12–14).

A cerebral abscess is an extremely rare but serious complication of esophageal dilation, with an incidence of 0.5% in pediatric patients (7, 15, 16).

Three different mechanisms for its development have been proposed, namely, contiguous spread (40%–50%), hematogenous dissemination (30%–40%), or cryptogenic (10).

In this context, a pathophysiological hypothesis has been proposed involving traumatic microinjuries or microperforations of the esophageal mucosa. This implies that dilation generates sufficient pressure to induce lesions and the subsequent spread of bacteria through the venous vessels. Once the bacteria reach the venous system, they can access the central nervous system through the Batson plexus or craniospinal system.

The Batson plexus is a valveless venous system described by Batson in 1940. This craniospinal venous red network links the thoracic, abdominal, pelvic, and spinal venous systems that permit bidirectional blood flow influenced by intra-thoracic or intra-abdominal pressure (17, 18).

Sudden pressure increases during coughing, Valsalva maneuvers, or esophageal instrumentation may reverse venous flow, enabling contaminated blood to bypass the pulmonary filter, reach the intracranial venous sinuses, and potentially lead to cerebral seeding (12, 18).

Cerebral abscesses evolve over approximately 14 days, progressing from cerebritis to a fibrous capsule (19, 20). Although rare, with 0.3 to 1.8 cases per 100,000 inhabitants per year, it carries high mortality across all age groups (21–24). Moreover, 25% of all cases affect children, usually those aged between 4 and 10 years (25–27).

Hematogenous abscesses are frequently localized in the frontal and parietal lobes (7, 28, 29). Clinical presentation includes headache (70%), fever (45%–53%), vomiting, and focal neurological deficits (48%–65%). Symptoms onset may be insidious, complicating the diagnosis. (7, 15). The definitive diagnosis is based on neuroimaging.

Viridans group streptococci, particularly *Streptococcus*, are commonly isolated and recognized for their propensity to cause abscess formation. *Streptococcus* colonization rates of 95%–98% have been documented in healthy individuals from the upper and lower esophageal tract. The most common species are *Streptococcus salivarius* (60%–65%), *Streptococcus mitis* (48%–50%), and the *Streptococcus anginosus* group (18%–20%) (30).

Since 1973, 23 pediatric patients and three adults have been reported with cerebral abscesses associated with esophageal dilation (see Table 1).

**Table 1.** 

Author	No. of cases	Age	Gender	No. dilations	Fever post-dilation	Days post-dilation	Brain region	Abscess culture	Cause
Rontal et al., 1973 ( 17 )	2	17 years	M	Multiple	No	56	Right temporal lobe	Not reported	Sodium and potassium hydroxide
Kotler et al., 1974 ( 28 )	3	1 year and 5 months	F	Multiple	Yes	180	Right frontal and temporal lobes	*Staphylococcus aureus* coagulase *+*	Sodium hydroxide
		2 years	F	Not reported	Not reported	150	Not reported	Not reported	Sodium hypochlorite
		1 year and 6 months	F	Multiple	Yes	270	Left frontal lobe	Not reported	Sodium hydroxide
Leahy et al., 1977 ( 29 )	2	6 years	M	Not reported	Yes	Not reported	Left temporal-parietal lobe	*Streptococcus group B* and *Staphylococcus epidermidis*	Sodium hypochlorite
		4 years	F	3	Not reported	Not reported	Right lateral lobe	*Staphylococcus aureus*	Sodium hypochlorite
Golladay et al., 1980 ( 13 )	1	6 years and 6 months	M	8	Yes	3	Left posterior temporal lobe	*Staphylococcus aureus*	Sodium hypochlorite
Gallegos et al., 1982 ( 31 )	1	6 years and 6 months	M	10	Not reported	Not reported	Left frontal-temporal lobe	*Haemophilus influenzae and Streptococcus pneumoniae*	Sodium hydroxide
Schlitt et al., 1985 ( 10 )	3	1 year and 5 months	M	2	Yes	95	Left parietal lobe	*Bacteroides melaninogenicus, Neisseria, and Peptococcus* sp*.*	Sodium hypochlorite
		3 years and 6 months	F	8	Yes	90	Right parietal lobe	*Bacteroides melaninogenicus, microaerophilic streptococcus*	Sodium hypochlorite
		4 years	M	3	Yes	215	Left frontal lobe	*Haemophilus paraphrophilus*	Sodium and potassium hydroxide
Neuman et al., 1986 ( 14 )	1	1 year and 4 months	M	9	Not reported	9	Right frontal-parietal lobe	*Streptococcus viridans*	Sodium hypochlorite
Bautista et al., 1988 ( 32 )	2	3 years	M	11	Yes	6	Left parietal lobe	Not reported	Sodium and potassium hydroxide
		1 year and 6 months	M	3	Not reported	21	Two right parietal occipital lobes	Not reported	Sodium and potassium hydroxide
Lui et al., 1988 ( 16 )	2	5 years	M	4	Not reported	7	Right parietal lobe	Sterile	Sodium hydroxide
		1 year and 4 months	M	7	Yes	Not reported	Multiple abscesses in both hemispheres	*Peptostreptococcus anaerobius*	Sodium hypochlorite
Djupesland et al., 1991 ( 33 )	1	62 years	F	25	Yes	8	Right frontal lobe	*Streptococcus milleri*	Sodium hydroxide
Lopez-Candel et al., 1993 ( 34 )	2	5 years	M	10	Yes	Not reported	Left temporal lobe	*Streptococcus viridans*	Sodium hydroxide
		3 years	M	19	No	Not reported	Right occipital lobe	*Streptococcus “m-g intermedius”*	Sodium and potassium hydroxide
Ersahin et al., 1995 ( 35 )	1	6 years	M	8	Not reported	Not reported	Left frontal and right parietal lobes	Not reported	Sodium hydroxide
Appignani and Trizzino, 1997 ( 36 )	1	2 years	Not reported	Multiple	Yes	15	Right frontal-parietal lobe	Not reported	Sodium hydroxide
Thapar et al., 2003 ( 37 )	1	23 years	F	5	Yes	Not reported	Multiple bifrontal abscesses	*Klebsiella*	Sodium and potassium hydroxide
Gaini et al., 2008 ( 38 )	1	59 years	F	22	Not reported	6	Left frontal lobe and thalamus	*Streptococcus alpha-hemolytic*	Sodium hydroxide
Hofmeyr et al., 2008 ( 39 )	1	2 years	M	4	No	Not reported	Left frontal and right parietal lobes	*Streptococcus milleri, Haemophilus aphrophilus*	Sodium and potassium hydroxide
Aslan et al., 2017 ( 40 )	1	8 years	M	Multiple	Not reported	15	Right temporal lobe	Sterile	Nitric acid
Welling et al., 2020 ( 41 )	1	7 years	M	4	Not reported	Not reported	Right frontal lobe	*Streptococcus viridans*	Sodium hydroxide

Among the 26 patients analyzed, only 19 reported the number of dilations, with an average of 8.6 dilations prior to the development of the abscesses. Post-dilation fever was documented in only 50% of the cases. In 16 cases, the average time between the last dilation and the diagnosis was 71.6 days.

A consistent finding among these cases was a history of multiple dilation procedures, suggesting a cumulative risk related to repeated mucosal injury. Although current guidelines do not recommend routine antibiotic prophylaxis for esophageal dilation, selective prophylaxis may be considered for patients requiring multiple procedures, given the high incidence of bacteremia and the severity of potential complications (12, 42).

In the present case, symptoms began 4 days after the last dilation. Considering that a mature cerebral abscess typically requires more than 15 days to evolve, the early onset of symptoms does not align with the expected temporal pattern. The timing suggests that bacterial seeding may have occurred during the procedure on 2 April because the patient developed hemoptysis afterward. As previously mentioned, the mean number of dilations in previous reports was 8.6 procedures. In our case, seven procedures were performed previously, with the patient presenting with a fever 24 h after the last one. Upon her initial hospitalization, the patient presented with hyponatremia, which was probably associated with cerebral salt-wasting syndrome and could be considered secondary to CNS complications, but this entity was not considered until the patient developed symptoms, delaying the diagnosis.

## Conclusions

Repeated esophageal dilation for caustic-induced strictures may predispose pediatric patients to rare but severe infectious complications, such as a cerebral abscess. Transient bacteremia combined with hematogenous dissemination through the Batson venous plexus provides a plausible pathophysiological explanation for this.

Clinicians should maintain a high index of suspicion for signs of intracranial infection in children who develop a fever or neurological symptoms after esophageal dilation, even weeks after the procedure. Early neuroimaging and prompt multidisciplinary management are critical.

Current knowledge is based primarily on case reports; therefore, in the absence of robust data, further research is needed to develop techniques that minimize mucosal trauma and reduce the risk of microperforations during dilation. It is crucial to standardize post-dilation neurological monitoring and obtain early neuroimaging when any neurological signs or a fever without a clear source are present.

Although managing the risks associated with the treatment of caustic-induced esophageal stricture is essential, the root of the problem remains in the accidental caustic ingestion. Esophageal stricture—and consequently, the entire cascade of complications—is preventable. The only way to reduce morbidity and mortality is through primary prevention of caustic ingestion.

## Data Availability

The original contributions presented in the study are included in the article/Supplementary Material, further inquiries can be directed to the corresponding author.
