# Supplementary material for: Cerebral abscess secondary to post-esophageal dilation bacteremia: A case report with a pathophysiological association to consider
Source: Front Pediatr. 2026 Apr 27;14:1805960. doi: 10.3389/fped.2026.1805960 (PMC13158208; doi:10.3389/fped.2026.1805960)
Supplement: Supplementary file 1 [file Presentation1.pptx]

## Slide 1
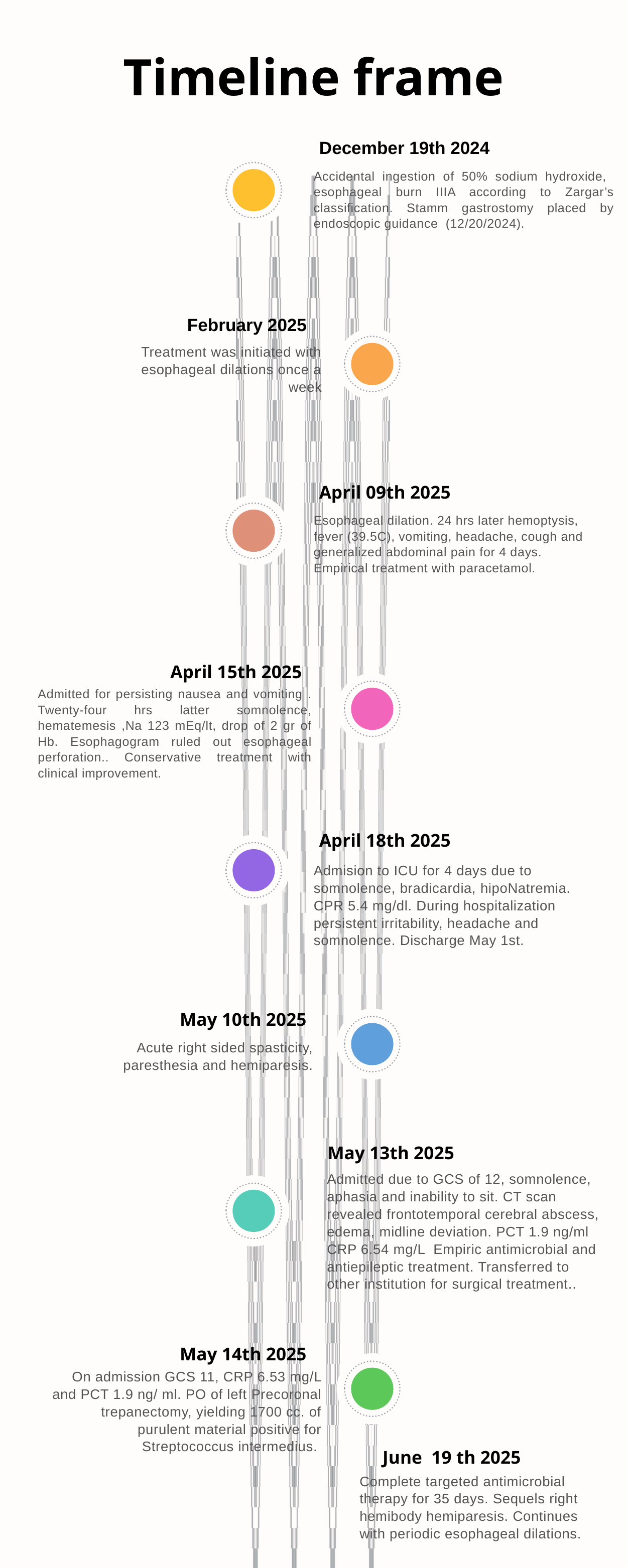

Timeline frame
December 19th 2024
Accidental ingestion of 50% sodium hydroxide, esophageal burn IIIA according to Zargar’s classification. Stamm gastrostomy placed by endoscopic guidance (12/20/2024).
February 2025
Treatment was initiated with esophageal dilations once a week
April 09th 2025
Esophageal dilation. 24 hrs later hemoptysis, fever (39.5C), vomiting, headache, cough and generalized abdominal pain for 4 days.
Empirical treatment with paracetamol.
April 15th 2025
Admitted for persisting nausea and vomiting . Twenty-four hrs latter somnolence, hematemesis ,Na 123 mEq/lt, drop of 2 gr of Hb. Esophagogram ruled out esophageal perforation.. Conservative treatment with clinical improvement.
April 18th 2025
Admision to ICU for 4 days due to somnolence, bradicardia, hipoNatremia. CPR 5.4 mg/dl. During hospitalization persistent irritability, headache and somnolence. Discharge May 1st.
May 10th 2025
Acute right sided spasticity,
 paresthesia and hemiparesis.
May 13th 2025
Admitted due to GCS of 12, somnolence, aphasia and inability to sit. CT scan revealed frontotemporal cerebral abscess, edema, midline deviation. PCT 1.9 ng/ml CRP 6.54 mg/L Empiric antimicrobial and antiepileptic treatment. Transferred to other institution for surgical treatment..
May 14th 2025
On admission GCS 11, CRP 6.53 mg/L and PCT 1.9 ng/ ml. PO of left Precoronal trepanectomy, yielding 1700 cc. of purulent material positive for Streptococcus intermedius.
June 19 th 2025
Complete targeted antimicrobial therapy for 35 days. Sequels right hemibody hemiparesis. Continues with periodic esophageal dilations.
